# Mechanical Dentistry

**Published:** 1851-04

**Authors:** T. L. Buckingham

**Affiliations:** Philadelphia.


### ARTICLE IX.

*Mechanical Dentistry.* By T. L. Buckingham, D. D. S., Philadelphia.

[Continued.]

After punching, cut the lining off the proper length and place it on the tooth. When all the linings have been punched and cut off, they should be filed to the shape required.

Some operators make the linings round on top, some square, and some oval on the outer surface; but, let the shape be what it may, they should all be of a length, and present an uniform appearance. When all are arranged they should be taken off, commencing at one end, and laying them in a row with the side up that goes next to the tooth; then put some borax on each lining, and also on the pins of the teeth; then replace the linings on the teeth, and with a sharp graver split each pin and wedge apart so as to bind the lining close to the tooth. The reason for placing borax between the linings and teeth, is, to aid in flowing the solder through the holes to the backs of the teeth. I have often found that when there was no borax there, the solder would only flow over the ends of the pins, and when I filed the solder off, the lining could be easily drawn off the tooth.

When the linings have all been fastened in this way, we may proceed to solder the whole case at once; but as it is very hard to finish up a case, when the teeth have been soldered to the lining and the lining to the plate all at one heat, I prefer to solder the lining to the tooth first. Remove the teeth from the plaster and lay them on a piece of charcoal with the lining up, borax the lining and lay a small piece of solder on each pin and heat them up very gradually; blow a broad, gentle flame, and move the charcoal so as to make the flame play in a circle around the teeth without touching them at first; then gradually diminish the circle until the flame comes directly on the teeth; still keep the charcoal moving until they are at a full red heat, then let the flame rest a moment on each tooth and the solder will flow. Great care should be taken in heating teeth; it should be done with a broad, steady, gentle flame. If they are heated with very fierce puffs of the blow-pipe, or with a jet of the flame, they are almost sure to break, and it is very annoying to have a tooth break when the case is so near done. When they are soldered, they should be covered at once with a piece of charcoal hollowed out, to protect them from the air in cooling. Now file the linings smooth and put the teeth back in their places in the plaster, but be careful that no pieces of plaster get into their places. When they are all in their proper position, mix some plaster and cover the points of the teeth as far down as the linings, which will keep them from being drawn out of their places in soldering to the plate.

If there be any places where the linings do not fit close to the plate, the space should be filled with small pieces of gold. Then borax well the joints to be soldered, and lay two or three pieces of solder on each joint. The solder now used should melt more readily than that used in soldering on clasps or linings to the teeth. The case is now ready for the last soldering. Commence blowing on the outside of the plaster, and move the charcoal so as to heat the plaster evenly all around; when the plaster is red hot on the outside, then bring the flame in on the plate and keep it moving from side to side until it is of a red heat, then let the flame rest on each tooth until the solder flows.

In soldering, the heat should be applied very gradually and steadily. The habit of using the blow-pipe properly is soon acquired, if we do not hurry too much. The difficulty in most cases is, that we blow too hard at first, and the lungs become exhausted before we are half through ; while, if we would blow gently, and when we found the lungs becoming exhausted, would stop and take an inspiration or two, we could get through without difficulty.

After the case is soldered, it should be let stand in the plaster until it is perfectly cold, for fear the cold air coming in contact with the teeth while hot, should break them. After being removed from the plaster, the case should be boiled out in some diluted sulphuric acid for a few minutes ; the teeth, however, being put in the acid before it is warmed. Where there is time to wait, let the case lay in the cold acid for thirty minutes ; this removes the borax and fire-coat from the plate. Then, with a graver or scraper and files, finish the case up as smooth as possible ; then with a strip of Scotch stone, stone all the file and other marks and scratches out of the plate, keeping it wet while stoning ; then wash all clean, and burnish with steel or blood-stone burnisher, using soap and water during the operation ; I prefer, however, to polish with a brush-wheel on a lathe, using sweet oil and tripoli or rotten stone ; then with a buff-wheel about the size of half a dollar, made of two or three thicknesses of hat-felt or thick buckskin, polish the plate all over where the buff will touch, and where it will not touch use a pointed stick covered with soft leather ; then brush it again with the brush-wheel, and wash clean with soap and water ; then with another buff-wheel made of soft buckskin on which use rouge, polish your plate well till you have a beautiful gloss ; wash off the rouge and dry the case with chamois leather or a soft napkin, or, what is better, cover the whole up in some dry saw dust for an hour or so, which will absorb the moisture without injuring the polish. The case is now ready for the mouth.—*Dental News Letter.*

